# Suspected Septic Sacroiliitis Following Recent Cesarean Section Under Spinal Anesthesia

**DOI:** 10.7759/cureus.8891

**Published:** 2020-06-28

**Authors:** Aratara A Nutcharoen, Ahmed A Salih, Andrew Volio, Alexis Skolaris, Sabry Ayad

**Affiliations:** 1 Anesthesiology, Cleveland Clinic Fairview Hospital, Cleveland , USA; 2 Outcomes Research, Cleveland Clinic , Cleveland, USA; 3 Outcomes Research, Cleveland Clinic, Cleveland, USA; 4 Anesthesiology, Cleveland Clinic Fairview Hospital, Cleveland, USA; 5 Anesthesiology, Cleveland Clinic, Cleveland, USA

**Keywords:** sacroiliitis, septic sacroiliitis, pyogenic sacroiliitis, sacroiliac joint, spinal anesthesia, cesarean section, mobility limitation

## Abstract

Septic sacroiliitis is sacroiliac (SI) joint inflammation secondary to microbial invasion of the synovial space characterized by tenderness over the sacroiliac joint, difficulty walking, and lower back pain that can radiate to the buttocks. Clinicians can easily overlook septic sacroiliitis as a potential diagnosis due to its rare occurrence and non-specific symptoms. A 30-year-old female (G2P2A0) who presented acutely to the ED nine days after an uncomplicated Cesarean section performed under spinal anesthesia. The patient experienced progressive lancinating, electric-shock pain originating from the coccyx radiating to the right buttock and lower back with the inability to bear weight. MRI pelvis demonstrated edematous changes of the right SI joint, indicative of septic sacroiliitis. Patient’s condition improved after empiric intravenous antibiotics and was discharged home on the continued intravenous course. Rapid empiric administration of intravenous antibiotics may have prevented the onset of severe complications of an infective SI joint.

## Introduction

Septic sacroiliitis is sacroiliac (SI) joint inflammation secondary to microbial invasion of the synovial space and is characterized by tenderness over the SI joint, difficulty walking, and lower back pain that can radiate to the buttocks [1]. Clinicians can easily overlook septic sacroiliitis as a potential diagnosis due to its rare occurrence and non-specific symptoms. Septic sacroiliitis has been associated with skin infections, IV drug use, trauma, rheumatoid arthritis, recent joint surgery, and joint prostheses [2]. The acute inflammatory reaction is caused by microorganism invasion of synovial tissue, which can lead to permanent destruction of the affected joint resulting in long-term decreased mobility [3]. We present an unusual case of severe pain originating from the coccyx radiating to the buttock and lower back with the inability to bear weight after an uncomplicated cesarean section performed under spinal anesthesia.

## Case presentation

A 30-year-old female G2P1A0 at 39w3d with no medical history was scheduled for an elective cesarean section under spinal anesthesia and levonorgestrel intrauterine device (IUD) placement which was placed prior to closure of the hysterotomy without complications. The patient had an uncomplicated peripartum course and delivered a healthy female infant. She received routine postpartum and postoperative care, ambulated without difficulty, and was discharged four days later.

Nine days after the uncomplicated cesarean section under spinal anesthesia, the patient presented to the ED and was admitted to the hospital for progressive lancinating, electric-shock pain originating from the coccyx, radiating to the right buttock and lower back. She reported 10/10 pain intensity with the inability to bear weight. No fever, neurologic deficits, paresthesia, weakness, or radiation down the legs were reported. Pain was exacerbated by walking and movement and alleviated when sitting and lying down.

**Figure 1:**
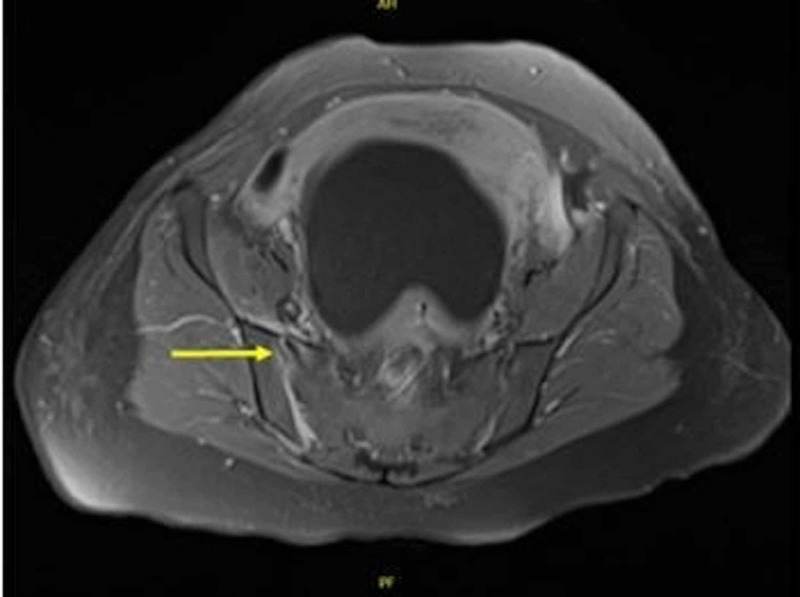
MRI pelvis demonstrating edematous changes (arrow) of the right sacroiliac (SI) joint, indicative of septic sacroiliitis

During the hospital course, the patient had elevated white blood cell (WBC), erythrocyte sedimentation rate (ESR), C-reactive protein (CRP), and C3 complement blood levels. MRI lumbar spine was unremarkable and ruled out spinal hematoma. An MRI pelvis demonstrated edematous changes of the right SI joint, indicative of septic sacroiliitis (Figure 1). CT-guided aspiration of the SI joint and bone biopsy were performed with negative findings. A sample aspirate was sent for polymerase chain reaction (PCR) assay, which showed no bacterial, viral, or fungal elements. Gram stain, bone biopsy, blood, tissue, and wound cultures were negative. Given clinical suspicion for septic sacroiliitis, the patient was treated empirically with IV ceftriaxone and vancomycin. Patient also received oral gabapentin, cyclobenzaprine, ketorolac, acetaminophen, ibuprofen, ondansetron, percocet, and lidoderm and diclofenac epolamine patches. The patient’s IUD was also removed on the first day of hospital admission.

The patient’s pain gradually improved and was well-controlled on oral medications. She was able to ambulate with a walker one week prior to discharge. The patient was discharged home 13 days after hospital admission with continued IV antibiotic administration through a peripherally inserted central catheter line for five weeks. In the follow-up visits, labs showed no inflammatory markers. Her pain significantly improved, and she was able to ambulate without a walker.

## Discussion

Septic arthritis is joint inflammation caused by an infectious etiology. Septic arthritis can be caused by bacterial (>95% all cases), fungal, parasitic, mycobacterial, viral, or other uncommon pathogens and is usually monoarticular [4,5]. The most frequently affected joint is the knee, followed by the hip, shoulder, and elbow [6]. The incidence of septic arthritis is rare, with between two and six cases per 100,000 people, with higher rates in pediatric populations or in patients with rheumatoid arthritis and endoprosthetic joints [4]. A subcategory of septic arthritis is septic sacroiliitis, which is a pyogenic infection of the sacroiliac joint with about 1%-2% of all cases affecting the SI joint [4]. If left untreated, some complications of septic sacroiliitis include rapid joint destruction, abscess formation, and osteomyelitis [7].

Clinical presentation of septic sacroiliitis consists of lower back or buttock pain, difficulty walking, low-grade fever (may or may not be present), tenderness, swelling, and painful limited range of motion [6,7]. Risk factors for developing septic sacroiliitis include skin infections, IV drug use, trauma, rheumatoid arthritis, recent joint surgery, and joint prosthesis [8]. The most common risk factor is preexisting joint disease or damage. Patients with rheumatoid arthritis are at increased risk for septic arthritis due to joint damage, poor skin condition, and immunosuppression [6]. Septic sacroiliitis frequently occurs in patients during the postpartum period. There is currently no relevant literature that states that there is any correlation b/w method of delivery and sacroiliac joint infections. However, a cesarean section may be the preferred method of delivery since it eases the pain on joints during labor [9].

Septic sacroiliitis can result from bacteremia due to the absence of a protective basement membrane within the joint lining allowing for bacterial entry into synovial fluid. Other causes may be direct inoculation from trauma or medical procedures and contiguous spread from osteomyelitis, abscess, cellulitis, or septic bursitis. The most common cause of sacroiliitis is Staphylococcus aureus, a gram-positive organism [6]. The majority of cases are due to gram-positive organisms with approximately 15% being due to gram-negative organisms. Small breaks in the skin and mucous membranes provide entry points for Gram-positive bacteria, while Gram-negative infections usually result from IV drug use, gastrointestinal sources, or urinary tract mucosal injury. Polymicrobial infections (i.e., Pantoea agglomerans and Nocardia asteroides) typically occur after penetrating trauma with foreign material or bite wounds [6]. Other organisms that may cause septic sacroiliitis include methicillin-resistant *Staphylococcus aureus*, *Pseudomonas aeruginosa*, *Staphylococcus epidermidis*, and *Escheria coli* [10].

Lower back and buttock pain is frequently observed during pregnancy and the postpartum period, therefore clinicians can easily overlook septic sacroiliitis as a potential diagnosis due to its non-specific symptoms. The hormonal effects of pregnancy can cause relaxation of ligaments supporting the sacrum, which can thin, stretch, and tear to cause bleeding or synovial joint effusion [11]. Diseases such as osteoarthritis, rheumatoid arthritis, gout, avascular necrosis, Lyme disease, malignancy, lumbar disc herniation, cellulitis, spinal abscess, urinary calculi, and pyelonephritis may mimic symptoms of septic sacroiliitis resulting in a challenging diagnosis [6,12].

There is no specific test for the diagnosis of septic sacroiliitis. Diagnosis usually depends on the history, physical exam, radiological imaging, and clinical judgment of an experienced physician. Serum blood tests may be ordered if there is clinical suspicion. In these cases, WBC, ESR, and CRP levels are elevated [13]. However, no study has demonstrated an acceptable sensitivity or overall diagnostic accuracy of peripheral WBC count for septic arthritis. Multiple studies demonstrated acceptable sensitivity for ESR of >30 mm/hour, but results were not consistent across trials, and when reported, specificities were uniformly poor [14].

Assessing synovial fluid for infectious organisms using arthrocentesis is currently the gold standard. Other diagnostic assessments include synovial fluid Gram stains and blood cultures [6]. However, a negative Gram stain does not by itself rule out septic arthritis since the sensitivity of Gram stains is poor, with 45% to 71% false-negative rates, its specificity remains undefined [14]. PCR assay may be used as a complementary tool rather than a diagnostic tool. A systematic review study indicated that PCR did not offer any advantage over bacterial cultures in the diagnosis of staphylococcal joint infections. Blood cultures may identify the causative organism even when synovial fluid cultures are unrewarding. Additionally, the absence of elevated WBC, ESR, or CRP does not exclude the diagnosis of septic sacroiliitis [15]. X-rays and ultrasounds are not usually helpful in diagnosing septic sacroiliitis. However, ultrasonography may be used to guide arthrocentesis. CT and MRI scans are considered more reliable diagnostic tools [7].

Treatment of septic sacroiliitis consists of using aggressive IV antibiotic therapy based on culture and sensitivity [7]. In cases where there is high suspicion for septic sacroiliitis, initial management consists of broad-spectrum IV antibiotics alongside culture-specific IV antibiotics and eventually a course of oral antibiotics. Transition from broad-spectrum to culture-specific antibiotics occurs after an adequate sample is aspirated from the SI joint or biopsy is performed. Non-steroidal anti-inflammatory drugs and muscle relaxants may be prescribed during the acute presentation, although these become less effective as cases become chronic [13]. Although some patients can be managed solely with antibiotics, recommended consultation with orthopedic surgery may suggest arthroscopy or arthrocentesis, which removes bacteria and toxins, decompresses the joint space, and improves blood flow to improve recovery [6]. Surgery is usually reserved as a last resort. These methods used with physical therapy can help treat septic sacroiliitis, alleviate pain, and strengthen lumbopelvic musculature [13].

In this case report, there was no conclusive diagnosis of what occurred in the SI joint; however, infection seemed most likely. Negative blood cultures and joint aspiration may be attributed to the following factors. Joint needle aspiration may have been in the wrong location; therefore the infectious organism may have been missed. Patient reported having a gluteal cleft dermal piercing forcibly removed two weeks prior to the development of symptoms, which may have been the initial source of infection. Three months prior, the patient was evaluated in the ED for a mechanical fall down ten steps on her lower back and buttocks which may have contributed to her pain. Additionally, the IUD insertion may have been another potential cause of infection, although it seems less likely.

## Conclusions

Permanent disability due to long-term joint damage and increased morbidity and mortality have been associated with delayed presentation, diagnosis, and treatment of septic sacroiliitis. Prompt diagnosis and rapid intervention are crucial to choosing the appropriate treatment and management for a successful recovery. Given the unusual presentation of this case, rapid empiric administration of IV antibiotics may have prevented the onset of severe complications of a septic sacroiliac joint.
